# How Are Inpatient Psychiatric Ward Rounds Understood in Research Literature? A Scoping Review

**DOI:** 10.1192/bjo.2024.223

**Published:** 2024-08-01

**Authors:** Benjamin Williams, Oluwatomilola Olagunju, Siobhan Richardson, Georgia Jameson

**Affiliations:** 1 Park House, Greater Manchester Mental Health NHS Foundation Trust, Manchester, United Kingdom; 2 Equality Diversity and Inclusion Team, Greater Manchester Mental Health, Manchester, United Kingdom

## Abstract

**Aims:**

Ward rounds are complex clinical interactions crucial in delivering high-quality, safe, and timely patient care. They serve as a platform for the multidisciplinary team to collaboratively assess a patient's condition and actively involve the patient and their caregivers in shared decision-making to formulate a care plan. Ward rounds involve an intersection of factors worthy of consideration separate from the wider literature on inpatient experience and multidisciplinary team meetings. With this review our primary aim is to systematically identify what methods and perspectives researchers are using to understand ward rounds.

**Methods:**

The databases searched were Medline, CINAHL, British Nursing Index, PsychInfo, and ASSIA as well as reference and citation checking. The search terms used were psychiatr* AND (*ward round* OR “*multi disciplinary team meeting*” OR “*clinical team meeting*”). Studies were included if they were peer reviewed, included primary research on psychiatric inpatient ward rounds in which patients are participants with no restriction on the type of ward or hospital, patient group, country or methodology.

**Results:**

224 records were retrieved and screened from the database search and 10 from other sources. 35 full texts were reviewed for eligibility and 26 included in the review. 16 studies had no particular theoretical perspective, 2 were constructivist, 2 critical realist, 2 lean methodology, 1 systems research, 1 phenomenological, 1 trauma informed and 1 critical theory. 9 focussed on patient experience, 5 ward round structure, 3 on power relationships, 3 on efficiency, 2 on shared decision making and 4 had a unique focus. Though often not explicit, critical theory influenced discussion of power is common in papers focused on patient experience and ward round structure. Cross-sectional surveys, interviews, focus groups and audit cycles were the most common methods. Key themes which emerge are anxiety provoked by ward rounds, preparation and communication, and the negotiation of power structures. Key tensions identified include being multidisciplinary versus overcrowding, efficiency versus personalisation and reliability versus responsiveness.

**Conclusion:**

For a central part of inpatient psychiatric practice there is a limited range of research on psychiatric ward rounds. The influence of critical theories’ focus on power was widespread with limited representation of other theoretical perspectives and concerns. There was no research using experimental methods, but there was some implementation research. Key tensions are highlighted which services may wish to consider when revisiting ways of working on inpatient psychiatric wards.

